# Citation distribution profile in Brazilian journals of general medicine

**DOI:** 10.1590/S1516-31802012000500008

**Published:** 2012-11-13

**Authors:** Luiggi Araujo Lustosa, Mario Edmundo Pastrana Chalco, Cecília de Melo Borba, André Eizo Higa, Renan Moritz Varnier Rodrigues Almeida

**Affiliations:** I BSc. Master’s Student, Biomedical Engineering Program (Coppe), Universidade Federal do Rio de Janeiro (UFRJ), Rio de Janeiro, Brazil.; II PhD. Associate Professor, Biomedical Engineering Program (Coppe), Universidade Federal do Rio de Janeiro (UFRJ), Rio de Janeiro, Brazil.

**Keywords:** Journal impact factor, Bibliometrics, Peer review, Systems for evaluation of publications, Health research evaluation, Fator de impacto de revistas, Bibliometria, Revisão por pares, Sistemas de avaliação das publicações, Avaliação da pesquisa em saúde

## Abstract

**CONTEXT AND OBJECTIVE:**

Impact factors are currently the bibliometric index most used for evaluating scientific journals. However, the way in which they are used, for instance concerning the study or journal types analyzed, can markedly interfere with estimate reliability. This study aimed to analyze the citation distribution pattern in three Brazilian journals of general medicine.

**DESIGN AND SETTING:**

This was a descriptive study based on numbers of citations of scientific studies published by three Brazilian journals of general medicine.

**METHODS:**

The journals analyzed were *São Paulo Medical Journal, Clinics* and *Revista da Associação Médica Brasileira*. This survey used data available from the Institute for Scientific Information (ISI) platform, from which the total number of papers published in each journal in 2007-2008 and the number of citations of these papers in 2009 were obtained. From these data, the citation distribution was derived and journal impact factors (average number of citations) were estimated. These factors were then compared with those directly available from the ISI *Journal of Citation Reports* (JCR).

**RESULTS:**

Respectively, 134, 203 and 192 papers were published by these journals during the period analyzed. The observed citation distributions were highly skewed, such that many papers had few citations and a small percentage had many citations. It was not possible to identify any specific pattern for the most cited papers or to exactly reproduce the JCR impact factors.

**CONCLUSION:**

Use of measures like “impact factors”, which characterize citations through averages, does not adequately represent the citation distribution in the journals analyzed.

## RESUMO

**CONTEXTO E OBJETIVO:**

O fator de impacto é atualmente o índice bibliométrico mais utilizado para a avaliação de revistas científicas. No entanto, as condições de seu uso, por exemplo, em relação ao tipo de estudos ou revistas analisadas, podem interferir significativamente com a confiabilidade de suas estimativas. Este trabalho teve como objetivo estudar o padrão de distribuição de citações em três periódicos brasileiros de medicina geral.

**TIPO DE ESTUDO E LOCAL:**

Trata-se de um estudo descritivo, baseado nos números de citações de trabalhos científicos publicados por três revistas brasileiras de Medicina Geral.

**MÉTODOS:**

As revistas analisadas foram *São Paulo Medical Journal, Clinics* e *Revista da Associação Médica Brasileira*. A pesquisa utilizou-se de dados disponibilizados pela plataforma do *Institute for Scientific Information* (ISI), da qual foram obtidos o total de publicações de cada revista no período de 2007-2008 e o número de citações a esses trabalhos em 2009. A distribuição de citações e os fatores de impacto (média do número de citações) para cada revista foram estimados a partir desses dados, e os fatores de impacto assim obtidos foram comparados aos fornecidos diretamente pelo ISI *Journal of Citation Reports* (JCR).

**RESULTADOS:**

No período, foram publicados respectivamente 134, 203 e 192 artigos pelas revistas analisadas. Observou-se que a distribuição das citações a esses artigos é altamente enviesada, com um grande número de trabalhos pouco citados e um pequeno percentual com muitas citações. Não foi possível identificar um padrão específico para os trabalhos mais citados ou reproduzir exatamente os fatores de impacto JCR.

**CONCLUSÃO:**

O uso de medidas como “fatores de impacto” que caracterizam citações por meio de médias não representa adequadamente a distribuição de citações nas revistas analisadas.

## INTRODUCTION

Impact factors (IFs) are currently the bibliometric index most used for evaluating scientific journals. This index is defined as the average number of citations received by the papers published in a journal, according to appropriately defined “time windows” (usually two years).^1^^,^^2^ They were originally developed by E. Garfield in 1955 and, since 1975, they have been published annually by the Institute for Scientific Information (ISI) (currently Thomson Scientific) in its *Journal of Citation Reports* (JCR).^2^

However, although widely used, the validity of IFs has been criticized, for example because of problems in defining them or in making comparisons between research fields and paper types.^3^^,^^4^^,^^5^^,^^6^ Another point of contention concerns their definition as averages, in a context in which medians would probably be a more adequate statistical index.^7^ On the other hand, few studies have tried to empirically assess the behavioral profile of published papers in scientific journals.

## OBJECTIVE

The objective of the present study was to investigate the distribution pattern of citations in three Brazilian journals of general medicine.

## METHODS

Three Brazilian journals of general medicine that are indexed on the ISI website were selected for this analysis: *São Paulo Medical Journal*, *Clinics* and *Revista da Associação Médica Brasileira (Rev Ass Med Bras)*. The papers published in these journals in 2007-2008 and the citations of them made in 2009 were identified from the ISI/Thomson Reuters (Web of Knowledge) platform.^8^ The data were analyzed using the R x642.12.2 software in Portuguese. The research field and type of paper (review article or original research) were also identified for the most cited articles in each journal.

The citation distributions for each journal were then recalculated, together with their medians. Finally, the impact factor of each journal was obtained in two ways: a) through the values available on the ISI/Web of Knowledge platform (in the JCR database); and b) by recalculating it from the “direct search” numbers, as described above. For this, the following expression was used:^2^

IF 2009 = NC2009/NA2007-2008 (1)

In the above expression, NA _[2007-2008]_ is the number of articles published in 2007-2008 and NC_2009_ is the number of citations of these articles in 2009 that were identified. The direct search was made on the printed or eletronic editions of each journal.

## RESULTS

For the *São Paulo Medical Journal,* 134 articles and 100 citations could be identified via ISI (IF = 0.746) versus 134 and 96 from direct search (IF = 0.72). For *Clinics,* 203 papers and 324 citations were found via ISI (IF = 1.596), while 203 papers and 288 citations were found from direct search (IF = 1.40). For the *Rev Ass Med Bras*, these numbers were 192 and 113 via ISI (IF = 0.589) *versus* 192 and 97 from direct search (IF = 0.50). These results are presented in Table 1.

**Table 1.** Numbers of articles, numbers of citations obtained via ISI, numbers of citations obtained from direct search and their medians, impact factor (IF) via ISI/JCR and IF recalculated from the direct search data, in *São Paulo Medical Journal, Clinics* and *Revista da Associação Médica Brasileira*, for articles published in 2007-2008 and cited in 2009

Journals	Articles (N)	Citations ISI (N)	Citations (direct search)		IF ISI	IF (direct search)
			(N)	(Median)		
São Paulo Medical Journal	134	100	96	1	0.746	0.72
Clinics	203	324	288	2	1.596	1.40
Revista da Associação Médica Brasileira	192	113	97	0	0.589	0.50

**Figure 1.**
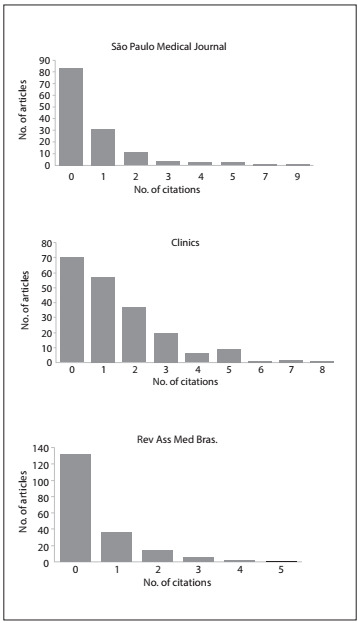
Distribution of citations (in 2009) of articles published in 2007-2008, in the journals *Clinics, Revista da Associação Médica Brasileira (Rev Assoc Med Bras)* and *São Paulo Medical Journal.* These citation numbers were obtained from direct searches in the ISI/Thompson Reuters platform.

In 2009, the three journals had similar citation distributions (Figure 1), with a high concentration of items with zero or close to zero citations (a positive skewness bias). The medians calculated for each journal are also listed in Table 1. The most cited articles received nine citations (*São Paulo Medical Journal*), eight (*Clinics*) and five (*Rev Assoc Med Bras*). In each journal, 95% of the citations were concentrated on 46 articles (*São Paulo Medical Journal*), 119 (*Clinics*) and 55 (*Rev Assoc Med Bras*). Out of these 220 articles (119 + 55 + 46), 18 were review papers.

## DISCUSSION

The objective of this commentary was to characterize the distribution profile of citations in three Brazilian journals of general medicine. From the results identified, the following points should be highlighted for discussion:

1. A high proportion of papers with few citations: As mentioned, most articles had zero or close to zero citations, while a small group of papers was highly cited, and in one of the journals analyzed, more than half of the published papers received zero citations during the period studied. The implications of this phenomenon are further outlined below.
2. Clearly skewed distribution: It follows from the above that the citation distributions obtained are not symmetrical. Under these conditions, it is well known that medians are better data description measurements.^7^ However, IFs are basically means (the mean number of citations of articles published over a certain period of time).
3. An arbitrary cutoff point (the “two-year” window) for calculating IFs: One explanation for the large number of items with zero citations is simply the arbitrary nature of the period traditionally used for calculating IFs (two years). If the time needed for planning, completion, submission and actual publication is taken into consideration, it is to be expected that, under typical circumstances, the real impact of a piece of research would only be perceived after a longer period. The ISI platform offers other time windows (five years and six months), but it is not possible to define an ideal size for these, and neither is it possible to suppose that this period would be the same for all journals, research lines or types of study. This adds an extra difficulty to calculating measurements such as IFs.
4. Inconsistency, albeit small, between the IFs estimated from direct search and those obtained via ISI: As said, it was not possible, in this study, to obtain the exact IF values that were available via ISI. This was due to inconsistencies in the number of citations attributed to the articles analyzed, although the same database was used. Similar phenomena have been commented on before^9^^,^^10^ and, in one case, the editors of a journal failed to reproduce the IF values for their own journal, even after obtaining access (presumably) to the same data used for the JCR calculation.^10^ This also creates a problem in using IFs, since calculating them becomes, ultimately, a process that is not fully open, and to which interested parties do not have complete access.
5. A small number of papers with a high number of citations: It is well known that factors that are not directly related to the degree of scientific innovation of a paper may influence its number of citations. For example, review articles usually receive more citations and have greater visibility than original research papers (as was the case of the most cited article in this study).^11^ In addition, research that is considered to be of “local interest” has less visibility, relative to research dealing with problems that are considered to be of “universal concern”.^12^ In the present study, among the articles that accounted for 95% of citations over the period studied, 18 were reviews.

IFs are an important index for scientific evaluation, but for their use to be effective, a number of issues still need to be resolved. For example, what is the optimal time for identifying the true impact of an article? Is it valid to classify journals ultimately according to outliers (papers with a very high number of citations)? Are measurements such as “averages” acceptable for assessing scientific impact? Is it acceptable for a community that is so conscious of the transparency of its methods to use procedures that are not fully reproducible? These issues are not resolved in this report: it only points out the fact that indicators such as IFs need more empirical research so that their limitations and conditions of use can be better specified and understood.

## CONCLUSION

The main conclusion from this analysis is that, for the three journals analyzed, the results pointed to skewed distributions, with a large number of articles with zero or close to zero citations. Moreover, despite using the same database, it was not possible to exactly reproduce the IFs that are provided by the ISI website.
